# Errors and discrepancies in the administration of intravenous infusions: a mixed methods multihospital observational study

**DOI:** 10.1136/bmjqs-2017-007476

**Published:** 2018-04-07

**Authors:** Imogen Lyons, Dominic Furniss, Ann Blandford, Gillian Chumbley, Ioanna Iacovides, Li Wei, Anna Cox, Astrid Mayer, Jolien Vos, Galal H Galal-Edeen, Kumiko O Schnock, Patricia C Dykes, David W Bates, Bryony Dean Franklin

**Affiliations:** 1 UCL Interaction Centre, University College London, London, UK; 2 Pain Management Centre, Imperial College Healthcare NHS Trust, London, UK; 3 Institute of Educational Technology, Open University, Milton Keynes, UK; 4 Research Department of Practice and Policy, UCL School of Pharmacy, London, UK; 5 UCL Medical School, University College London, London, UK; 6 Royal Free London NHS Foundation Trust, London, UK; 7 Faculty of Computers and Information, Cairo University, Cairo, Egypt; 8 Brigham and Women’s Hospital, Boston, Massachusetts, USA; 9 Harvard Medical School, Boston, Massachusetts, USA; 10 Department of Medicine, Brigham and Women’s Hospital, Boston, Massachusetts, USA; 11 Centre for Medication Safety and Service Quality, Imperial College Healthcare NHS Trust, London, UK

**Keywords:** medication safety, patient safety, information technology

## Abstract

**Introduction:**

Intravenous medication administration has traditionally been regarded as error prone, with high potential for harm. A recent US multisite study revealed few potentially harmful errors despite a high overall error rate. However, there is limited evidence about infusion practices in England and how they relate to prevalence and types of error.

**Objectives:**

To determine the prevalence, types and severity of errors and discrepancies in infusion administration in English hospitals, and to explore sources of variation, including the contribution of smart pumps.

**Methods:**

We conducted an observational point prevalence study of intravenous infusions in 16 National Health Service hospital trusts. Observers compared each infusion against the medication order and local policy. Deviations were classified as errors or discrepancies based on their potential for patient harm. Contextual issues and reasons for deviations were explored qualitatively during observer debriefs.

**Results:**

Data were collected from 1326 patients and 2008 infusions. Errors were observed in 231 infusions (11.5%, 95% CI 10.2% to 13.0%). Discrepancies were observed in 1065 infusions (53.0%, 95% CI 50.8% to 55.2%). Twenty-three errors (1.1% of all infusions) were considered potentially harmful; none were judged likely to prolong hospital stay or result in long-term harm. Types and prevalence of errors and discrepancies varied widely among trusts, as did local policies. Deviations from medication orders and local policies were sometimes made for efficiency or patient need. Smart pumps, as currently implemented, had little effect, with similar error rates observed in infusions delivered with and without a smart pump (10.3% vs 10.8%, p=0.8).

**Conclusion:**

Errors and discrepancies are relatively common in everyday infusion administrations but most have low potential for patient harm. Better understanding of performance variability to strategically manage risk may be a more helpful tactic than striving to eliminate all deviations.

## Introduction

Intravenous medication administration is complex, and data suggest that errors are common. For example, a systematic review of nine studies across various stages of intravenous medication preparation and administration reported errors in 73% of intravenous doses.^1^ However, published error rates vary widely, from 18% to 173% of intravenous doses in studies using structured observation of medication administration.^2^

Amidst concerns over safety, technologies such as ‘smart pumps’ have been advocated. These incorporate dose error reduction software to check programmed infusion rates against preset limits within a customisable drug library. However, dose limits can be over-ridden, and evidence regarding their impact is mixed.^3 4^ While unintended infusion overdoses represent a major safety concern, there are many factors that affect infusion administration, and smart pumps are just one possible solution.

A recent multisite US study using structured observation reported a high prevalence of intravenous infusion administration errors and procedural failures, even with the use of smart pumps, yet few potentially harmful errors.^4^ Building on this and an earlier US study,^5^ we therefore wanted to conduct a similar study in the UK with a larger sample size^6^ to confirm or refute these findings in a different context in which smart pumps are less common. In contrast to previous studies, we also wanted to incorporate a Safety II approach to interpret our findings.^7^ This approach moves away from the traditional focus of classifying all deviations as errors and blaming the human for unreliable processes. Instead it encourages consideration of deviations in terms of performance variability, how to understand and manage this variability, and that the human component can make positive contributions to safety.^7 8^ Our objectives were to determine the prevalence, types and severity of errors and discrepancies in intravenous infusions in England and to explore sources of variation, including the potential contribution of smart pumps, using a Safety II approach.

## Methods

### Study design

We used a point prevalence observational study of intravenous infusions in a sample of hospitals, followed by debriefs with staff at each site to gather additional context. Although we built on previous studies,^4 5^ we did not consider all deviations from the medication order or local policy to be errors: minor or intentional deviations were classed as discrepancies. The study protocol was published previously^6^ and the study was approved by a National Health Service (NHS) Research Ethics Committee (14/SC/0290).

### Study setting and sample

We used a purposive sampling strategy to select 16 NHS trusts in England, aiming for a diverse range of organisations in terms of type, size, location, patient safety metrics and use of infusion devices and smart pump technology.^6^ Online supplementary appendices 1 and 2 summarise the recruitment process and characteristics of each participating trust. We conducted observations in three clinical areas (general medicine, general surgery and critical care) in 13 trusts; in eight of these we also conducted observations in paediatrics and oncology day care. Two specialist children’s hospitals collected paediatric data only. One further trust collected oncology day care data at three hospital sites. We aimed to include a sample of 2100 infusions across all participating sites to give a CI around a 10% error rate of 8.7%–11.3%.^6^

10.1136/bmjqs-2017-007476.supp1 Supplementary data

### Data collection

Data were collected between April 2015 and December 2016. At each trust, two observers (usually a nurse and a pharmacist) employed in the organisation were trained by the research team to collect data. This training included highlighting the types of deviations to look for, conducting observations in the presence of the research team where possible and using sample cases to facilitate discussion about classification of deviations identified. Observers were also requested to identify and familiarise themselves with relevant local policies and guidelines prior to data collection. Observers then spent 1 weekday or equivalent collecting data in each clinical area. One clinical area could comprise one or more wards. Observers aimed to collect data on all intravenous infusions being administered at the time of data collection, including drugs, fluids, blood products and nutrition. Bolus doses were excluded, except where a prescribed bolus was given as an infusion, or vice versa. Completed infusions were excluded even if still attached to the patient. Patients were not observed if they were in isolation due to infection risks, were receiving care that would have required interruption or were off the ward.

Observers compared each medication being administered against the prescription and local policies/guidance,^6^ and consulted clinical staff if needed to understand any deviations. Data were recorded using a standardised paper form and subsequently uploaded to a secure web-based tool.^9^ No patient identifiable data were recorded. Suspected errors were raised with clinical staff so they could be corrected if needed; local reporting practices were then followed.

### Identifying and assessing deviations

We recorded any deviations from a prescriber’s written or electronic medication order, the hospital’s intravenous policy and guidelines, or the manufacturer’s instructions. We included the administration of medication to which the patient had a documented allergy or sensitivity, but did not assess other aspects of the clinical appropriateness of the medication order. We also collected data on policy violations and procedural or documentation deviations that may increase the likelihood of medication administration errors occurring. These included patients not wearing an identification wristband with the correct information, medication or infusion administration sets not being labelled in accordance with hospital policy and failure to document the administration of medication in line with policy. Finally, we encouraged observers to record any other irregularities, anomalies or workarounds related to the administration. Some of these were grouped together for analysis and formed new categories. Online supplementary appendix 3 presents definitions of deviation types.

10.1136/bmjqs-2017-007476.supp2 Supplementary data

**Figure 1:**
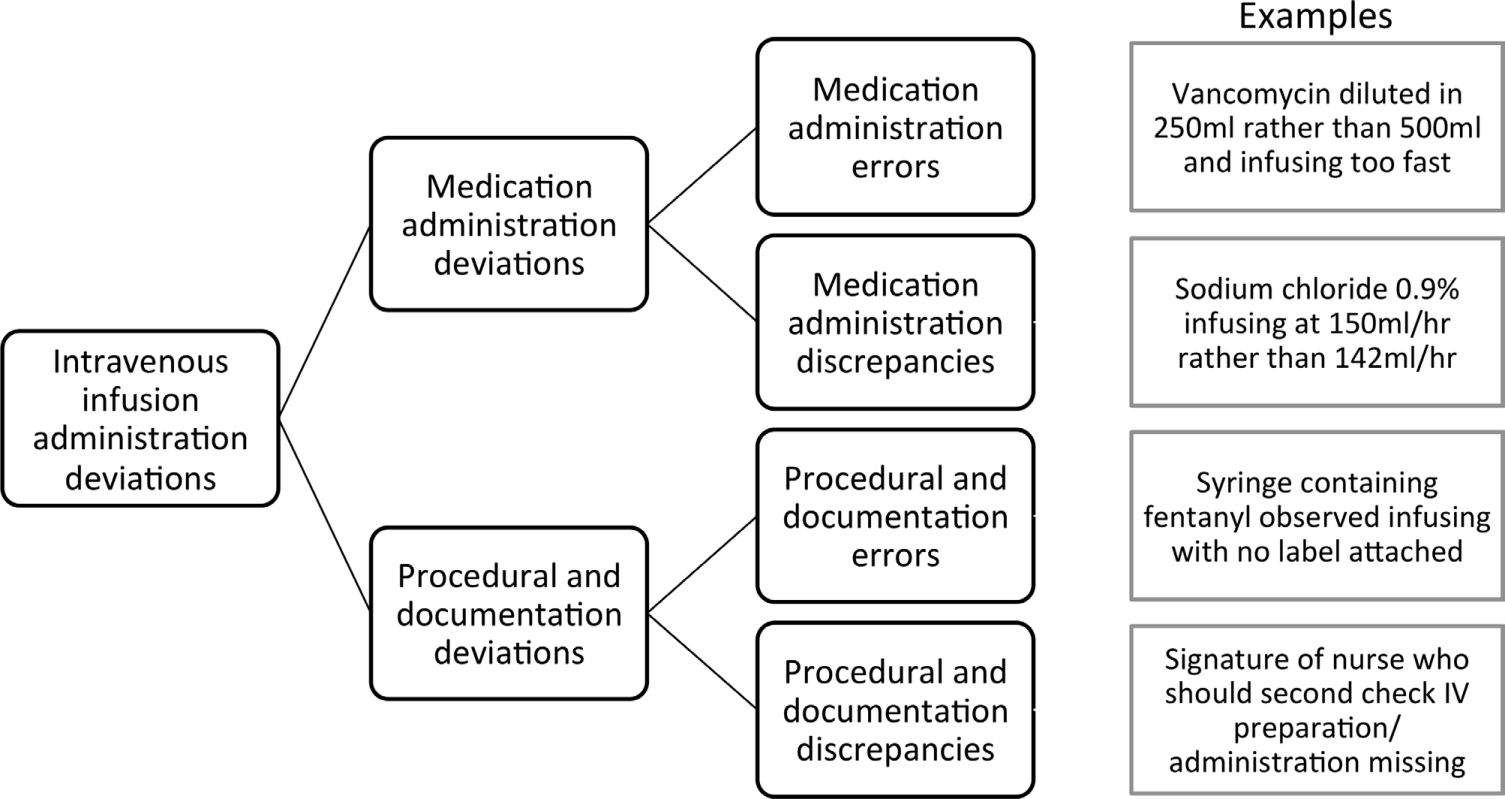
Classification of deviations, errors and discrepancies.

Local observers rated each deviation using an adaptation of the National Coordinating Council for Medication Error Reporting and Prevention (NCCMERP) severity index.^10^ Ratings were based on the likelihood of the deviation resulting in patient harm if it had not been intercepted, and were used to classify the deviations as discrepancies (rated A1 or A2) or errors (rated from Cto I) (online supplementary appendix 4).^6^ Based on these ratings we developed and clarified our classifications, recognising that deviations could be either errors or discrepancies, either in medication administration or in the associated procedural and documentation requirements (figure 1). We report separately on a comparison between the NCCMERP ratings and an alternative severity classification method based on expert judgement.^11^

Observers at each trust documented brief descriptions of any deviations identified and provided further qualitative insights during semistructured debriefs once data collection was complete.

### Data management and analysis

Clinicians within the research team reviewed deviations that had uncertain classifications, for example, where local observers highlighted that they found categorisation difficult and where observers had classified similar deviations differently. Clinicians within the research team also reviewed each error rated category D (‘likely to have required increased monitoring and/or intervention to preclude harm’) and above. Minor changes were made to classifications of type and severity of error as needed.

Error and discrepancy rates were calculated as the proportion of infusions with at least one error or discrepancy using total opportunities for error (total number of doses administered, plus any omitted doses) as the denominator. Results are presented according to overall error and discrepancy rates, and individual types of errors and discrepancies, grouped into medication administration deviations, and procedural and documentation deviations. Variations in deviation rates between clinical areas, delivery modes and infusion types were explored descriptively with their 95% CIs, and Χ^2^ tests where appropriate. Qualitative data were analysed inductively.

## Results

Overall, 6491 patients were present in the clinical areas observed, of whom 1545 (23.8%) were receiving and/or prescribed an intravenous infusion at the time of data collection. Data were collected from 1326 (85.8%) patients, who were administered and/or prescribed 2008 infusions.

### Frequency, types and potential severity of errors and discrepancies

Overall, 240 errors and 1491 discrepancies were identified across 2008 intravenous infusions. Table 1 presents the numbers and percentages of infusions and patients affected. Table 2 shows the types of deviations observed and their likely harm. Ninety per cent of observed errors were considered unlikely to cause harm despite reaching the patient (NCCMERP category C). Twenty-two errors (9.5%) were category D, and one (0.4%) category E; these 23 potentially harmful errors represent 1.1% of infusions. Examples in each severity category are presented in table 3.

**Table 1:** Number and proportion of infusions and patients with at least one error or discrepancy

	Infusions (n=2008)		Patients (n=1326)	
	One or more errors (ie, Cto I severity ratings) n (%; 95% CI)	One or more discrepancies (ie, A1 and A2 severity ratings) n (%; 95% CI)	One or more errors (ie, Cto I severity ratings) n (%; 95% CI)	One or more discrepancies (ie, A1 and A2 severity ratings) n (%; 95% CI)
Medication administration deviations	207 (10.3; 9.1% to 11.7%)	241 (12.0; 10.6% to 13.5%)	197 (14.9; 13.0% to 16.9%)	222 (16.7; 14.8% to 18.8%)
Procedural or documentation deviations	24 (1.2; 0.8% to 1.8%)	953 (47.5; 45.3% to 49.7%)	23 (1.7; 1.1% to 2.6%)	694 (52.3; 49.6% to 55.1%)
Miscellaneous	5 (0.2; 0.1% to 0.6%)	13 (0.6; 0.3% to 1.1%)	5 (0.4; 0.2% to 0.9%)	13 (1.0; 0.6% to 1.7%)
All deviations*	231 (11.5; 10.2% to 13.0%)	1065 (53.0; 50.8% to 55.2%)	219 (16.5; 14.6% to 18.6%)	781 (58.9; 56.2% to 61.6%)

*Some infusions were affected by more than one type of discrepancy; therefore the number and percentage of infusions affected by at least one error or discrepancy of any type is not the sum of each deviation type.

**Table 2:** Number, frequency and potential severity of each type of deviation

Type of deviation	Errors				Discrepancies		
	NCCMERP severity rating			n (% of 2008 infusions)	NCCMERP severity rating		n (% of 2008 infusions)
	C	D	E		A1	A2	
Medication administration deviations							
Rate deviation	65	12	–	77 (3.8)	48	27	75 (3.7)
Unauthorised medication	72	3	–	75 (3.7)	–	1	1 (0.0)
Administration start time discrepancy	13	–	–	13 (0.6)	31	8	39 (1.9)
Incomplete or delayed completion	10	–	–	10 (0.5)	4	27	31 (1.5)
Expired drug	11	–	–	11 (0.5)	1	1	2 (0.1)
Dose discrepancy	5	2	–	7 (0.3)	6	6	12 (0.6)
Wrong drug/fluid/diluent	11	–	–	11 (0.5)	1	1	2 (0.1)
Omitted medications (not administered at time of data collection)	2	3	–	5 (0.2)	1	6	7 (0.3)
Roller clamp positioned incorrectly or inappropriately	1	–	–	1 (0.0)	–	10	10 (0.5)
Concentration discrepancy	–	–	1	1 (0.0)	7	2	9 (0.4)
Drug library not used or incorrectly used (in the case of smart pumps)	–	–	–	–	–	67	67 (3.3)
Allergy oversight	–	–	–	–	2	–	2 (0.1)
All medication administration deviations	190	20	1	211	101	156	257
Procedure or documentation deviations							
Infusion administration set not tagged/labelled correctly	–	–	–	–	–	537	537 (26.8)
Documentation of the administration	1	–	–	1 (0.0)	–	334	334 (16.6)
Additive label missing or incorrect	16	1	–	17 (0.8)	2	200	202 (10.1)
Patient identification*	6	–	–	6 (0.3)	–	110	110 (5.5)
Documentation of the medication order	–	–	–	–	7	31	36 (1.8)
All procedure or documentation deviations	23	1	–	24	9	1212	1219
Miscellaneous	4	1	–	5 (0.2)	4	9	13 (0.6)
All deviations	217	22	1	240	114	1377	1491

*Deviations are counted per infusion; this figure includes patient identification deviations (ie, no name band) applied to all infusions for those patients. There were 88 patient identification discrepancies, counting each once per patient.

NCCMERP, National Coordinating Council for Medication Error Reporting and Prevention.

**Table 3:** Examples of observed deviations in the administration of intravenous infusions

Severity category	Examples
E	Patient was administered 2 g vancomycin diluted in 250 mL of sodium chloride 0.9%. The drug should have been diluted in 500 mL of sodium chloride 0.9% (concentration error: severity category E) and administered over at least 240 min. The drug was observed running too fast via gravity feed (rate error: D). The chart had not been signed to confirm the administration had been double-checked as required (documentation discrepancy: A2). The patient suffered from pain and red lumps along arm.
D	Piperacillin/tazobactam was prescribed to be given over 3 hours. However, it was given as a bolus over 3–5 min, which is the most common way to administer this antibiotic. The nurses presumed the doctors had made a mistake and corrected it. However, this had been prescribed intentionally after discussions with the consultant, with microbiology, with pharmacy and the drug manufacturer due to the patient’s poor renal function. This clinical decision was recorded in the patient’s notes but nursing staff had not reviewed these. 40 mmol of potassium chloride rather than the prescribed 20 mmol was administered together with 10 mmol magnesium sulfate in sodium chloride 0.9% at 1000 mL/hour.
C	1 L sodium chloride 0.9% with potassium chloride 0.15% was prescribed over 12 hours. The documented start time was 23:25. When observed at 13:00 the following day the infusion was not running and approximately 150 mL remained. The infusion should have been complete but the pump was not plugged in and the battery was empty. A medication order for 20 mcg fentanyl stated diluent as dextrose 5%, however the drug was prepared and administered in sodium chloride 0.9%.
A2	Electronic prescription specified 1 L of sodium chloride 0.9% over 8 hours. Started at 02:00 thus due to finish 10:00 but at 09:25 there was still 500 mL to run. The infusion was paused at the time of observation as the patient was receiving an intermittent amoxicillin infusion. Hartmann’s solution had been selected in the smart pump’s drug library but the infusion being administered was sodium chloride 0.9% (at the correct rate prescribed).
A1	The prescribed rate was 250 mL/hour for 123 mg paclitaxel in 250 mL sodium chloride 0.9%. However, the final reconstituted volume was 290.5 mL, which was being infused at 290 mL/hour to give the same rate of administration as prescribed. Administration of piperacillin/tazobactam was delayed by approximately 30 min.

#### Medication administration deviations

Overall, 427 (21.3%) infusions involved at least one medication administration error (n=211) or discrepancy (n=257). The most frequent types of deviation concerned rates and unauthorised medications.

##### Rate deviations

Overall, 152 infusions (7.6%) were being administered at a different rate from that prescribed; 77 were classified as errors (rated ≥C) and 75 as discrepancies (rated A1 or A2). A large proportion involved order changes that had not been correctly documented and infusions titrated based on the patient’s clinical need or fluid allowance without such titration being prescribed. Three deviations involved prescribed boluses administered as infusions, and one was a prescribed infusion given as a bolus.

About 31% of rate errors occurred in infusions delivered via gravity (without using a pump), despite accounting for just 8% of infusions. Of the 12 most serious rate errors (rated D), eight were administered via gravity; these included red blood cells, vancomycin, paracetamol and piperacillin/tazobactam. Many medication orders specified a duration rather than rate (eg, over 8 hours). In one case an infusion was observed running at a very high rate to ‘catch up’—1 L of Plasmalyte 148 had been prescribed over 24 hours; at the time of observation, 27 hours after the start time, the rate was set at 500 mL/hour.

##### Unauthorised medication

Eighty-nine infusions did not have a corresponding medication order. Thirteen were flushes that did not require a medication order according to local policy. Therefore, 76 infusions (3.8%; 75 errors, one discrepancy) were judged to be unauthorised. Almost half were fluids used to flush the line, commonly in oncology settings, including sodium chloride 0.9% (n=29), dextrose (1), Plasmalyte (2) and heparin (3). A further seven infusions were sodium chloride 0.9% administered at low rates to keep the vein open. Twenty infusions were unauthorised repeats of previously prescribed maintenance fluids. Four were administered based on verbal orders that had not been documented at the time of observation. Of the remaining 10 unauthorised infusions, seven involved maintenance fluids and three were drugs (calcium folinate, remifentanil, insulin). The remifentanil infusion had been prescribed and subsequently discontinued, but not represcribed after a decision to resedate the patient.

#### Procedural and documentation deviations

Overall, 961 infusions (47.9%) had at least one procedural or documentation error (n=24) or discrepancy (n=1219). Table 2 shows the frequency and severity of different types of procedural and documentation deviations. Non-compliance with hospital requirements for labelling infusion administration sets was most common. Procedural or documentation errors mostly involved unlabelled syringes, or infusions where the label was significantly inaccurate. For example, a patient prescribed 60 mg pamidronate was being administered an infusion labelled as 30 mg, but staff confirmed the patient had received the correct dose.

While some of the discrepancies identified in our study were deviations from protocols that may have been intentional workarounds, this was not always the case. Some were minor, non-clinically significant variations from what was prescribed that did not meet our definition of a medication administration error (eg, small deviations in flow rate or concentration, or minor delays to maintenance fluids’ start or finish times due to being interrupted to administer intravenous antibiotics), and some were minor documentation discrepancies.

### Sources of variation in error and discrepancy rates

**Figure 2:**
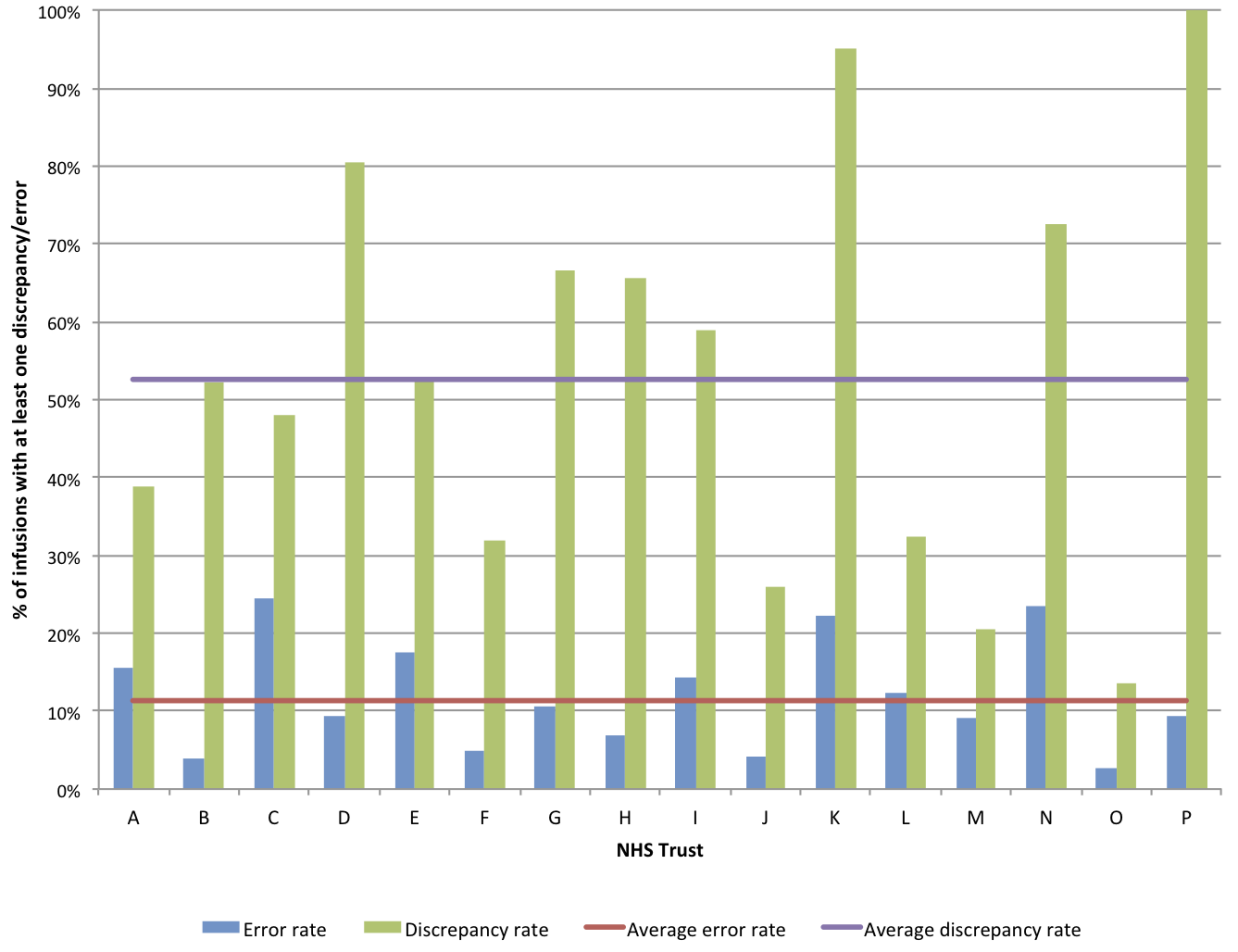
Variation in error and discrepancy rates between National Health Service (NHS) trusts.

Error rates among trusts ranged from 2.7% to 24.4%, and discrepancy rates from 13.5% to 100% of infusions, with no evidence of a relationship between error and discrepancy rates (figure 2). Procedural or documentation deviations ranged from 9.9% to 100% of infusions across trusts, reflecting wide variation in hospital policies and how they were enacted in practice. Some trusts had stringent policy requirements (eg, trust K) whereas others did not (eg, trust J); some had requirements that staff were unaware of in practice (eg, trusts D and P).

Variation was also evident among clinical areas and different infusion types (online supplementary appendix 5). Infusions observed in critical care had a lower error rate (7.0%); the error rate for paediatric areas was similar to that for adult non-critical care areas. Patient-controlled analgesia pumps and syringe drivers had the lowest error rates at 6.4% and 5.1%, respectively, with infusions delivered via gravity the highest (21.5% of 163 infusions). Error rates also varied by type of medication; maintenance fluids (eg, sodium chloride 0.9%) had a high error rate (18.5%) compared with drugs (6.9%), blood products (9.1%) and parenteral nutrition (2.9%).

Eleven of 16 hospitals (69%) used smart pumps (ie, an infusion pump with a drug library and/or dose error reduction software enabled) in at least one clinical area. However, just 640 (32%) infusions were administered using a smart pump (online supplementary appendix 5). Infusions delivered using smart pumps had similar error rates to those using other pumps (10.3% vs 10.8%; p=0.8). No appropriate entry was available in the drug library for one-third of infusions administered using a smart pump. Of 424 infusions with a library entry available, the library was used in 356 (84%) cases. There was no significant difference in error rates for doses given via a drug library versus those given without (online supplementary appendix 5). Discrepancy rates were higher in infusions delivered using smart pumps (61.7% of 640 infusions) compared with those without smart features (46.4% of 1202 infusions, p<0.001). Sixty-seven discrepancies were identified in the use of a smart pump drug library: 61 where the drug library was bypassed completely and six where the wrong entry was selected. However, differences in discrepancy rates were more commonly linked with policy requirements for labelling infusions and administration sets at different sites; when discrepancies related to use of a drug library are excluded, the discrepancy rate remains higher in infusions delivered via smart pump (59.2% of 640 infusions).

### Qualitative insights

Information provided by observers revealed some reasons for deviations. Some were simple slips or lapses such as confusing diluents and forgetting to open roller clamps to start the infusion; others involved lack of knowledge of policy requirements. Staff also reported deliberate deviations that would benefit patients but conflicted with official rules and formal procedures, for example, giving patients fluids that had not yet been prescribed when a doctor was unavailable (unauthorised fluids) and keeping lines patent by switching to a low infusion rate in anticipation of another infusion being needed (rate deviation). There were several instances of inaccurate prescriptions that were ‘corrected’ and administered by nurses without getting the order changed prior to administration. However, in one case the administering nurse incorrectly assumed an unusual prescription was wrong (table 3—piperacillin/tazobactam).

In some instances, nursing staff actively tried to balance risk and efficiency rather than follow procedures mechanistically. For example, staff reported stopping infusions (delay in completion) when patients left the ward for investigations so a nurse did not have to accompany the patient when staffing resources were stretched. In addition, some nurses objected to spending time labelling administration sets and writing batch numbers on additive labels for short infusions that would soon be discarded.

Observers at some trusts reported that collecting the study data provided insights into the reasons for some deviations and helped them identify solutions. For example, at one site where poor compliance with documentation of medication administration was recorded, the trust subsequently purchased handheld computers to allow staff to access electronic records in closer proximity to patients.

## Discussion

We found that 1 in 10 intravenous infusions involved an error, and one in two involved a discrepancy. However, few were considered likely to cause patient harm. There was considerable variability in errors, discrepancies, policies and practices among trusts. Our mixed methods approach offers insights into some reasons for this variability. Nurses can be a source of resilience, compensating for deficiencies and vulnerabilities in the system; however, this same adaptive capacity can also lead to unsatisfactory outcomes.^12^ Informed by Safety II, 7 8 our findings suggest the need to question traditional notions of ‘error’ and the goal of eliminating all errors and discrepancies. Instead we reflect on a broader notion of deviations, highlight positive contributions to efficiency and safety that go beyond compliance and explore strategic interventions to manage performance variability.

### Disentangling errors, discrepancies and harm

Overall, we found a lower error rate (11.5%) than that reported in much research into intravenous medication error (range 35%–85.9%).^13–15^ Some of this difference can be explained by methodological differences, for example, inclusion of bolus doses and preparation errors in other studies. The difficulties in comparing error rates between studies using different methods and definitions, in different contexts, have been well documented.^15 16^ Comparing studies using similar methods,^4 5^ we found broadly comparable rates of potentially harmful errors, with errors rated D or above in 1.1% of infusions in our study, and 0.4%^4^ and 3.8%^5^ elsewhere. We also identified similar error types, with the most common medication administration errors being rate deviations and unauthorised medications, and the most common procedural and documentation deviations concerning labelling of medication and administration sets. However, our overall error rate remains lower than in these studies, probably due to our more nuanced distinction between errors and discrepancies.

While several studies consider the potential harm associated with errors and some distinguish between medication administration errors and procedural failures or policy violations,^4 13^ we are not aware of previous studies that sought to understand the context of the deviation by distinguishing errors and discrepancies. Researchers and practitioners may have differing views on what constitutes an error,^17^ with a range of situations identified that clinicians may not consider errors.^18 19^ These judgements are largely ignored in definitions of errors adopted in most previous studies. Separation of discrepancies and errors in our study allowed us to better capture the complexities of current intravenous practices, and may be more acceptable to clinicians who feel that the realities of practice mean that policies cannot always be adhered to.

Previous studies have highlighted the importance of procedural failures and policy violations in identifying system weaknesses that may create latent conditions for patient harm.^5^ In this study, we recognise that both medication administration and procedural/documentation deviations occur on a spectrum from minor discrepancies to serious errors with potential for harm. While severe errors naturally attract greater attention, and are often the focus for intervention, a Safety II perspective encourages us to look at ‘normal’ discrepancies to identify potential system weaknesses. According to Safety II, people make adjustments to respond to the demands of the situation and compensate for system weaknesses. We identified several cases where these adjustments avoided or mitigated potential harm. However, these same adaptive mechanisms can also lead to unsatisfactory outcomes, as identified in one instance in this study. A challenge for safety management is that everyday discrepancies appear trivial but can contribute to rarer and more serious incidents.^20^ Our approach to distinguishing discrepancies and errors may help clinicians to reflect on different kinds of deviations, consider which are important and identify discrepancy patterns that may be concerning.

### Policy and practice gaps

Much of the variability among trusts related to gaps between policy and practice. Better understanding of the reasons behind such performance variability is necessary to target interventions that improve safety. Procedural and documentation deviations may not always represent poor practice but rather a poor fit between official policy and everyday practice due to situational constraints. In some cases, policies that better reflect existing practice may be more beneficial in managing risk to both patients and staff than enforcing compliance with existing policy. For example, policies allowing administration of flushes without a medication order in specific circumstances or for specific patient groups, already in place in many trusts, could be introduced at hospitals where unprescribed flushes are accepted local practice by clinical staff but are technically unauthorised. National standardisation may be helpful for whether or not small volume flushes need to be prescribed and if so how, labelling requirements for intravenous infusions and giving sets, and requirements for double-checking.

### Implications for practice: strategic interventions and smart pumps

Appreciating the nuances of frequency, types and severity of deviations occurring in different contexts moves us beyond interventions focused on improving compliance and eliminating error, towards more strategic interventions to proactively manage risk. Care is rarely delivered in ideal circumstances and a more pragmatic and practical approach, incorporating a wider range of strategies, is needed.^8^ Strategic decisions to live with certain deviations might be made if efforts to resolve them are likely to distract from other aspects of patient care, or not translate into gains for patient safety. More work is needed to understand if and how routine performance variability in intravenous infusions can spiral into rare and unsatisfactory outcomes, what conditions contribute to poor outcomes and which interventions should be prioritised to prevent harm rather than only reducing discrepancies.

Smart pumps are one possible intervention to improve safety in intravenous infusion administration. Similar to previous US studies,^4 5^ we found that smart pumps, as currently implemented in English hospitals, do not seem to reduce the risk of error in everyday practice. Although smart pumps may have a role in preventing more severe and rare errors, our relatively limited observation periods did not identify these. In addition, greater attention to the configuration and usability of pumps is required: a third of smart pumps used in our study offered no advantage over standard pumps due to incomplete drug libraries. Using smart pumps as part of an integrated system with bar code scanning and interfacing with electronic systems could guard against a broader range of deviations. Although the costs and benefits of implementing such a system have not yet been established,^4 5^ such approaches have become standard practice in the USA as both were included in the government’s Meaningful Use programme, which provides financial incentives to promote the use of health information technologies to improve quality. Such configurations are rare in English hospitals; no participating trusts used bar code administration, and only a minority had trust-wide electronic prescribing and medication administration records. The high error rate associated with infusions delivered without a pump in our study suggests that efforts to reduce reliance on gravity feed, where it is difficult to control the delivery rate, may be a more immediate and achievable priority than the expansion of smart pump technology.

### Strengths and limitations

This was a large multisite study, incorporating hospitals with widely differing medication processes and systems, reflecting the diversity of intravenous infusion practices within the English NHS. Adopting a mixed methods approach provided a rich understanding of intravenous medication errors and the contexts in which they occur. There are advantages and disadvantages of using local observers versus observers from a research team. Employing local data collectors may have allowed less conspicuous observation and reduced the likelihood of nurses modifying their behaviour on observation days. However, using local staff may have resulted in some interobserver variability or institutional blindness to local poor practice. Variability was minimised as much as possible by using two observers from different professional backgrounds at each site where possible, providing training, and subsequent review of data by the multidisciplinary research team. Resource limitations and confidentiality agreements precluded measurement of interobserver reliability across sites.

Other limitations are acknowledged. The timing of data collection at each trust depended on local approvals and staff availability; both daily and seasonal variation in staffing levels and workload may have affected deviation rates. We focused on infusions running at the time of observation and will therefore have underestimated the overall medication administration error rate; observation of prescribing, dispensing, preparation and setting up infusions is likely to have revealed further errors.^21^ Errors already identified and corrected by smart pumps or a double check by another staff member prior to our observations would also not be captured using our methodology. Ward managers were aware the study was investigating medication administration errors and discrepancies, so it is possible that nurses changed their behaviour on observation days. However, observation dates were not publicised in advance and nurses were not directly observed, thus the impact is likely minimal. Finally, our study was not powered to test for associations between pump and infusion types and error rates; our findings instead highlight areas for further investigation.

## Conclusion

Overall, we identified errors in 1 in 10 infusions, but very few were likely to result in patient harm. Smart pumps, as currently implemented, seemed to have little effect, with similar error rates observed in infusions delivered with and without a smart pump. Measuring the prevalence, types and severity of errors and discrepancies can provide valuable insights for reflection. However, this needs to be coupled to causal accounts and contextual understanding of local hospital policies, cultures, customs and practices. Not all deviations from medication order or policy are bad; many arise as nurses actively manage safety and productivity pressures. This study suggests there is a need to shift the focus away from the goal of eliminating deviations to enable strategic intervention to manage infusion risk in the context of everyday performance variability and working conditions. Future work should explore where efforts should be targeted to prevent harm rather than only reducing discrepancies.
