# Supplementary figures and images for: Errors and discrepancies in the administration of intravenous infusions: a mixed methods multihospital observational study
Source: BMJ Qual Saf. 2018 Apr 7;27(11):892–901. doi: 10.1136/bmjqs-2017-007476 (PMC6225796; doi:10.1136/bmjqs-2017-007476)

Appendix 1: Recruitment Flow Chart

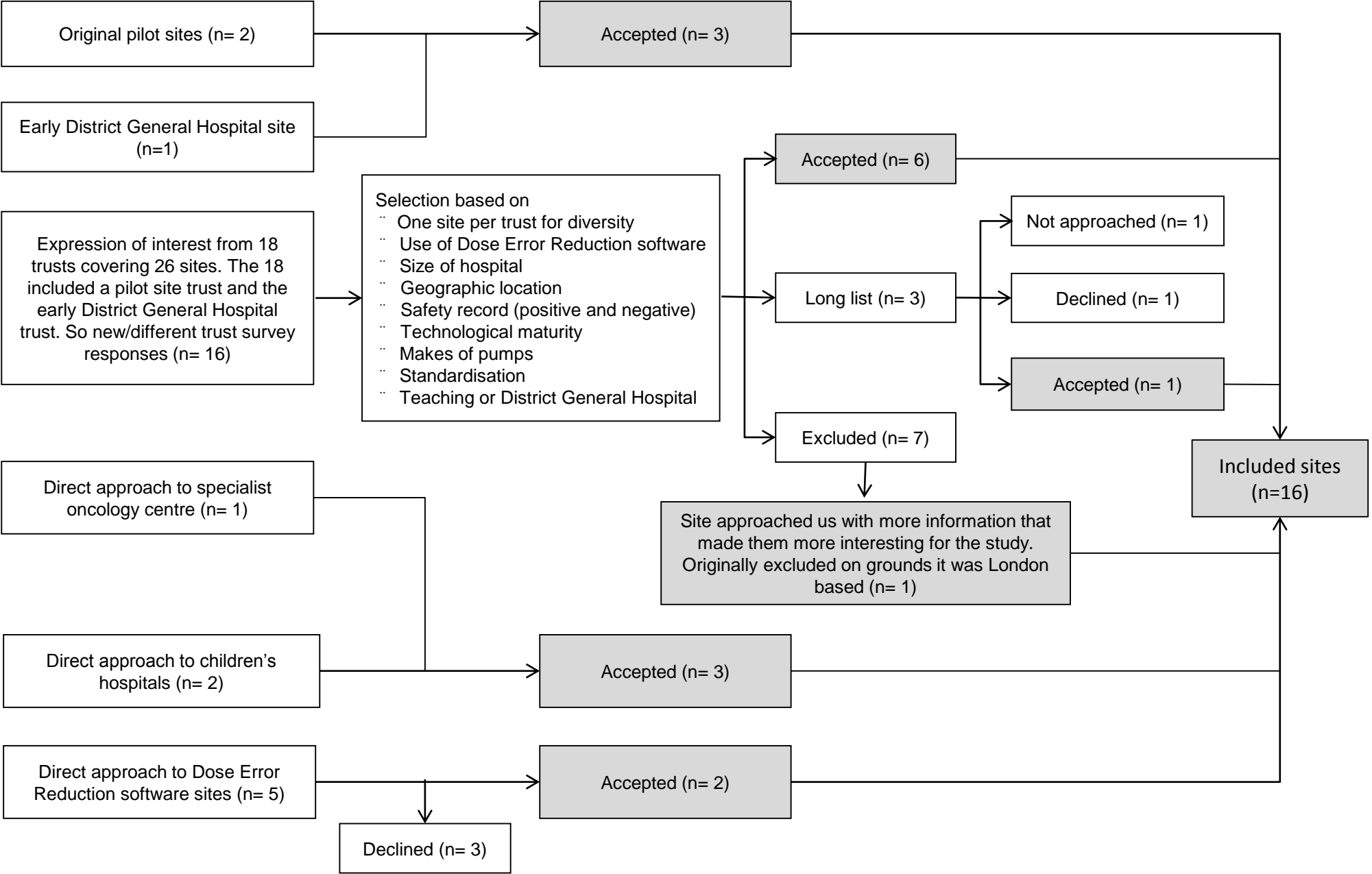

Supplement: Supplementary data [file bmjqs-2017-007476supp001.pdf]
