# Supplementary material for: Errors and discrepancies in the administration of intravenous infusions: a mixed methods multihospital observational study
Source: BMJ Qual Saf. 2018 Apr 7;27(11):892–901. doi: 10.1136/bmjqs-2017-007476 (PMC6225796; doi:10.1136/bmjqs-2017-007476)
Supplement: Supplementary data [file bmjqs-2017-007476supp002.pdf]

Appendix 2: Participating Site Characteristics

Trust	A	B	C	D	E	F	G	H	I	J	K	L	M	N	O	P
Region	London	London	South	South	South	South	South	North	Midlands	South	London	North	London	London	London	North
Hospital Type	TH	TH	DGH	DGH	DGH	DGH	DGH	DGH	CH	DGH	TH	CH	TH	TH	OC	TH
Number of beds (approximate)	500	850	350	505	380	550	575	760	360	290	1000	150	600<br>400	850	270	750
Clinical areas included	GM, GS, CC	GM, GS, CC, P, O	GM, GS, CC, P, O	GM, GS, CC, P, O	GM, GS, CC	GM, GS, CC, P, O	GM, GS, CC	GM, GS, CC, P, O	P	GM, GS, CC	GM, GS, CC, P, O	P	GM, GS, CC, P, O	GM, GS, CC, P, O	O	GM, GS, CC
Days of data collection	3	5	5	5	3	8	3	5	6	3	5	5	5	5	6	5
Data collection period	Apr-May 2015	Jun-Oct 2015	Aug 2015	Jun-Dec 2015	Aug 2015	Sep 2015 - Mar 2016	Jun-Oct 2015	Sep 2015	Aug 2015 - Mar 2016	Sep 2015	Feb-Apr 2016	Oct 2015 -Feb 2016	Mar-May 2016	Jan 2016	Aug-Dec 2016	Sept-Oct 2016
Variety of pump brands used	Brand 1<br>Brand 4<br>Brand 9	Brand 2<br>Brand 8	Brand 2<br>Brand 4<br>Brand 9	Brand 2<br>Brand 5	Brand 6	Brand 4	Brand 1<br>Brand 2<br>Brand 7<br>Brand 9	Brand 1<br>Brand 4<br>Brand 9	Brand 3<br>Brand 4<br>Brand 8	Brand 1<br>Brand 2	Brand 4<br>Brand 7	Brand 4<br>Brand 5	Brand 4<br>Brand 7	Brand 2<br>Brand 10	Brand 1<br>Brand 9	Brand 2<br>Brand 5<br>Brand 7
Clinical areas in which smart pumps used (pump types)	None	CC (Vol, Syr)	CC (Vol, Syr)<br>GS (PCA) P (PCA)	CC (Vol, Syrr)<br>GS (PCA)	None	None	CC (Vol, Syr)<br>GM (Vol, Syr)<br>GS (Vol)	CC (Vol, Syr)<br>GM (Vol)	CC (Syr)<br>GM (Vol, Syr)<br>GS (Syr)	All (PCA)	All	All (Syr)	All areas	GS (PCA)	None	CC<br>GM<br>GS
Computerized physician order entry	None*	Some areas	Some areas (O)	Some areas	Some areas (CC, GM)	None	None	Some areas (O, GS)	Some areas	None	Some areas	None	Some areas (O)	Yes	Some areas (O)	Yes

TH, Teaching Hospital; DGH, District General Hospital; CH, Specialist Children's hospital; OC, Specialist Oncology Centre

GM, General medicine; GS, General surgery; CC, Critical care; P, Paediatrics; O, Oncology day care

Vol, Volumetric pump; Syr, Syringe Driver; PCA, PCA pump

*Implemented after our data collection

Appendix 3: Definitions of deviation types

**Types of deviation**	**Definition**
Medication administration deviations (errors and discrepancies)	
Unauthorised medication/fluids (no documented order)	Fluids/medications are being administered but no medication order is present. This includes failure to document a verbal order if these are permitted as per hospital policy.
Wrong medication or fluid	A different fluid/medication/diluent as documented on the IV bag (or bottle/syringe/other container) is being infused compared with that specified on the medication order or in local guidance.
Concentration discrepancy	An amount of a medication in a unit of solution that is different from that prescribed.
Dose discrepancy	The same medication but the total dose is different from that prescribed.
Rate discrepancy	A different rate is being delivered from that prescribed. Also refers to weight-based rates calculated incorrectly including using a different patient weight from that recorded on the patient's chart.
Delay of dose or medication/fluid change	An order to change the medication or rate not carried out within 4 hours of the written medication order, or as per local policy.
Omitted medication or IV fluids	The medication prescribed was not administered.
Allergy oversight	Medication is prescribed / administered despite the patient having a documented allergy or sensitivity to the drug concerned.
Expired drug	The expiry date / time on either the manufacturer's or additive label has been exceeded.
Roller clamp discrepancy	The roller clamp is not positioned appropriately/ correctly.
Incomplete infusion or delayed completion*	
Procedural and documentation deviations (errors and discrepancies)	
Patient identification error	Patient either has no identification (ID) band on wrist, or information on their ID band is incorrect.
Wrong or missing information on additive label	Any incorrect or missing information on the additive label, as required by hospital policy
Giving set not tagged according to policy	Tagging or labelling of giving set is different (either missing or incorrect) from requirements in hospital policy
Documentation of the medication administration	Medication/fluids administered but not documented correctly on chart e.g. missing signature, start time, etc.
Documentation of the medication order*	Medication/fluids administered based on an incomplete, poorly documented or ambiguous medication order e.g. missing signatures or dates, the absence of a specific time to be administered where required, or the absence of clear instructions that a medication should be titrated to clinical need or within certain parameters.

***Category added during analysis phase based on 'other' discrepancies and errors**

Appendix 4: Adapted NCC MERP Index

Harm	Category	Description
No Error	A1	Discrepancy but no error
	A2	Capacity to cause error
Error, no harm	B	An error occurred but is unlikely to reach the patient
	C	An error occurred but is unlikely to cause harm despite reaching the patient
	D	An error occurred that would be likely to have required increased monitoring and/or intervention to preclude harm
Error, harm	E	An error occurred that would be likely to have caused temporary harm
	F	An error occurred that would be likely to have caused temporary harm and prolonged hospitalization
	G	An error occurred that would be likely to have contributed to or resulted in permanent harm
	H	An error occurred that would be likely to have required intervention to sustain life
Error, death	I	An error occurred that would be likely to have contributed to or resulted in the patient's death

Appendix 5: Variation in error and discrepancy rates

		Number of infusions	At least one error per infusion (i.e., NCCMERP B to I ratings) with 95% confidence interval	At least one discrepancy per infusion (i.e. A1 and A2 ratings)	At least one deviation per infusion
**Clinical area**	General medicine	366	50 (13.7%; 10.2 - 17.2%)	244 (66.7%)	261 (71.3%)
	Paediatrics	342	45 (13.2%; 9.6 – 16.8%)	171 (50.0%)	183 (53.5%)
	General surgery	402	51 (12.7%; 9.4 to 16.0%)	228 (56.7%)	244 (60.7%)
	Oncology day care	386	49 (12.7%; 9.4 – 16.0%)	180 (46.6%)	182 (47.2%)
	Critical care	512	36 (7.0%; 4.8 – 9.2%)	242 (47.3%)	250 (48.8%)
**Delivery method**	Gravity feed	163	35 (21.5%; 15.2 – 27.8%)	109 (66.9%)	119 (73.0%)
	Other (e.g. transducer/pressure bag	25	4 (16.0%; 1.6 – 30.4%)	8 (32.0%)	10 (40.0%)
	Volumetric pump	1364	164 (12.0%; 10.3 – 13.7%)	723 (53.0%)	759 (55.6%)
	Syringe driver	375	24 (6.4%; 3.9 – 8.9%)	187 (49.9%)	193 (51.5%)
	Patient controlled analgesia pump	78	4 (5.1%; 0.2 – 10.0%)	35 (44.9%)	36 (46.2%)
**Smart pump**	Pump with no smart features enabled	1202	130 (10.8%; 9.0 – 12.6%)	558 (46.4%)	592 (49.3%)
	Smart pump	640	66 (10.3%; 7.9 – 12.7%)	395 (61.7%)	406 (63.4%)
	Smart pump used with drug library	356	31 (8.7%; 5.8 – 11.6%)	241 (67.7%)	244 (68.5%)
	Smart pump drug library not used, although relevant drug listed	67	11 (16.4%; 7.5 – 25.3%)	67 (100%)*	67 (100%)*
	Smart pump drug library not used, as relevant drug not listed	215	23 (10.7%; 6.6 – 14.8%)	85 (39.5%)	93 (43.3%)
	Not known	2	0	0	0
**Infusion type**	Fluid	829	153 (18.5%; 15.9 – 21.1%)	476 (57.4%)	510 (61.5%)
	Blood or blood product	55	5 (9.1%; 1.5 – 16.7%)	13 (23.6%)	15 (27.3%)
	Drug	1012	70 (6.9%’ 5.3 – 8.5%)	507 (50.1%)	524 (51.8%)
	Parenteral nutrition	102	3 (2.9%; 0 – 6.2%)	59 (57.8%)	61 (59.8%)

* Not using the drug library was included as a discrepancy, hence 100% discrepancy rate
